# Myocardial bridging of the left anterior descending coronary artery as a risk factor for atrial fibrillation in patients with hypertrophic obstructive cardiomyopathy: a matched case–control study

**DOI:** 10.1186/s12872-021-02185-1

**Published:** 2021-08-06

**Authors:** Changrong Nie, Changsheng Zhu, Qiulan Yang, Minghu Xiao, Yanhai Meng, Shuiyun Wang

**Affiliations:** 1 Department of Cardiovascular Surgery, Fuwai Hospital, National Center for Cardiovascular Diseases, Chinese Academy of Medical Sciences and Peking Union Medical College, Beilishi Road 167, Xicheng District, Beijing, 100037 China; 2 Department of Intensive Care Unit, Fuwai Hospital, National Center for Cardiovascular Diseases, Chinese Academy of Medical Sciences and Peking Union Medical College, Beijing, China; 3 Department of Ultrasound, Fuwai Hospital, National Center for Cardiovascular Diseases, Chinese Academy of Medical Sciences and Peking Union Medical College, Beijing, China

**Keywords:** Myocardial bridging, Atrial fibrillation, Hypertrophic cardiomyopathy, Hypertrophic obstructive cardiomyopathy

## Abstract

**Background:**

Myocardial bridging (MB) is associated with various forms of arrhythmia. However, whether MB is a risk factor for atrial fibrillation (AF) in patients with hypertrophic obstructive cardiomyopathy (HOCM) remains unknown. This study aimed to identify the relationship between myocardial bridging of the left anterior descending coronary artery (MB-LAD) and AF in patients with HOCM.

**Methods:**

We reviewed the medical records of 1925 patients diagnosed with HOCM at Fuwai Hospital from January 2012 to March 2019. Patients with coronary artery disease, a history of heart surgery, and those who had not been subjected to angiography were excluded. Finally, 105 patients with AF were included in this study. The control group was matched in a ratio of 3:1 based on age and gender.

**Results:**

Forty-three patients were diagnosed with MB-LAD in this study. The presence of MB was significantly higher in patients with AF than in those without AF (19.0% vs. 7.3%; *p* = 0.001), although MB compression and MB length did not differ between the two groups. In conditional multivariate logistic analysis, MB (odds ratio [OR] 2.33; 95% confidence interval [CI] 1.08–5.01; *p* = 0.03), pulmonary arterial hypertension (OR 2.63; 95% CI 1.26–5.47; *p* = 0.01), hyperlipidemia (OR 1.83; 95% CI 1.12–3.00; *p* = 0.016), left atrial diameter (OR 1.09; 95% CI 1.05–1.13; *p* < 0.001), and interventricular septal thickness (OR 1.06; 95% CI 1.003–1.12; *p* = 0.037) were independent risk factors for AF in patients with HOCM.

**Conclusions:**

The presence of MB is an independent risk factor for AF in patients with HOCM. The potential mechanistic link between MB and the development of AF warrants further investigation.

## Background

Myocardial bridging (MB) refers to a congenital coronary abnormality, characterized by an epicardial coronary artery that passes intramurally through the myocardium, resulting in compression of the tunneled segment on coronary angiography during systole. This congenital variation usually involves the left anterior descending coronary artery (LAD). When MB was first identified by coronary angiography, most investigators considered this anomaly to be a benign condition. Many studies have suggested that MB is associated with various forms of arrhythmias, such as supraventricular tachycardia, ventricular tachycardia, atrioventricular conduction block, and even sudden cardiac death [1]. However, most of these studies are case reports, and clinical studies examining the relationship between MB and arrhythmia are limited.

Hypertrophic cardiomyopathy (HCM) is a common inherited cardiac disease with a prevalence of approximately 0.2–0.5% in the general population [2]. Among patients with HCM, the prevalence of MB is surprisingly high, with a prevalence of up to 30% [3]. Moreover, atrial fibrillation (AF) is the most common sustained arrhythmia in patients with HCM, with an annual incidence of up to 2–3% and a lifetime prevalence approximately 20–30%, which is associated with significant symptoms, functional decline, increased thromboembolic risk, and mortality [4, 5]. The relationship between MB and AF in patients with hypertrophic obstructive cardiomyopathy (HOCM) remains unknown.

## Methods

### Study aim

The purpose of this single-center retrospective study was to identify the relationship between MB-LAD and AF in patients with HOCM.

### Population

We retrospectively studied 105 patients with HOCM complicated with AF and 315 patients with HOCM that did not have AF out of a cohort of 1925 patients who were diagnosed with HOCM at Fuwai Hospital in Beijing between January 2012 and March 2019. The control group (HOCM patients without AF, N = 315) and HOCM patients with AF group (N = 105) were matched in a ratio of 3:1 based on age and gender. Patients who had coronary artery disease, had undergone heart surgery, or had no angiography records were excluded.

## Definitions

### Hypertrophic obstructive cardiomyopathy

The diagnostic criteria for HOCM were referred to the 2011 American Heart Association/American College of Cardiology guideline [6], which primarily included unexplained septal hypertrophy with a thickness of > 15 mm or septal myocardial thickness of > 13 mm with a family history of HCM. Moreover, HCM patients with an LVOT gradient of > 50 mmHg at rest or with provocation were considered to have left ventricular outflow tract (LVOT) obstruction.

### Atrial fibrillation

The clinical diagnosis of AF was based on electrocardiography, 24 h Holter electrocardiography, or in-hospital electrocardiogram monitoring. Paroxysmal AF refers to episodes that spontaneously returns to sinus rhythm within 7 days or with interventions, and may reoccur with variable frequency. Persistent AFs are episodes lasting > 7 days, which do not return to sinus rhythm without the intervention of a physician [7]. In this study we divided AF into two categories according to the definition of AF described above.

### Pulmonary arterial hypertension

Pulmonary arterial hypertension (PH) was defined as pulmonary artery systolic pressure (PASP) of > 35 mmHg without pulmonary stenosis or right ventricular outflow tract obstruction [8].

### Invasive coronary angiography

**Fig. 1:**
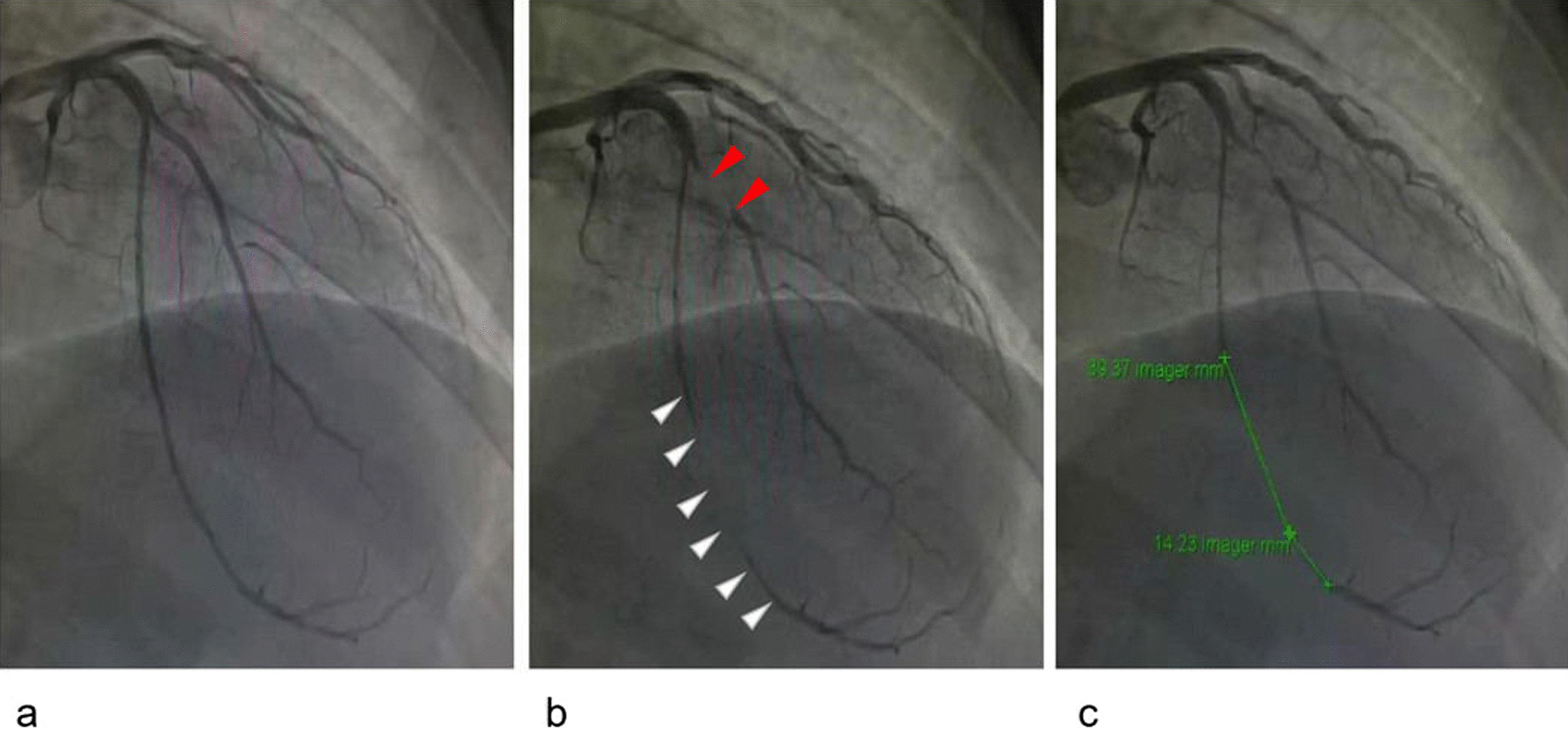
Myocardial bridging (MB) demonstrated by invasive coronary angiography during diastole (**a**) and systole (**b**). There was a significant narrowing on the anterior descending coronary artery during systole (white arrows). The MB on the diagonal branch (red arrows) or any other branch was rare and not analyzed in this study. The length from the beginning to the end of the coronary artery narrowing was measured as MB length (**c**)

All patients in this study underwent coronary angiography using a standard procedure. When a MB was found, the location, length and maximal degree of dynamic narrowing of the MB were clearly described (Fig. 1). In this study, isolated MB was defined as a reduction of > 30% in the diameter of the mural artery during systole without any significant atherosclerosis. All angiograms were assessed by an experienced doctor who was unaware of the patients’ clinical status.

### Echocardiography

Transthoracic echocardiographic studies were performed using a commercially available system (E9 ultrasound system, GE Healthcare, Horten, Norway). All images were analyzed by applying standard measurements according to the guidelines of the American Society of Echocardiography and using the echocardiograph’s internal quantitative package. The diameters of the cardiac chambers were presented as the maximum values of the anteroposterior diameter in cardiac cycles. Maximum left ventricular wall thickness was the greatest dimension measured at any site within the left ventricular chamber at end-diastole. The LVOT gradient was scanned with continuous-wave Doppler to measure maximal outflow velocity and estimated using the simplified Bernoulli equation. More details can be found in our previous publication [9].

### Statistical analysis

Continuous variables were expressed as mean ± standard deviations (SD), and categorical variables were presented as number (percentage), as appropriate. We used Student’s t-test to compare independent samples and the χ2 or Fisher exact tests to compare nominal variables. Propensity score matching was used to balance age and gender between the HOCM with AF and the HOCM without AF groups. The propensity score was calculated via a logistic regression model with 1:3 nearest neighbor matching without replacement, and the caliper width was set to 0.2. Univariate and stepwise multivariate conditional logistic regression analyses were used to select the factors associated with AF. Variables with a *p*-value of < 0.1 in univariate analysis were entered into the multivariate analysis. All p-values were two-tailed, and *p*-values of < 0.05 indicated statistical significance. R 3.6.0, SPSS version 26.0, and GraphPad Prism version 8.0 were used for calculation and illustration, respectively.

## Results

### Baseline patient characteristics

Out of the 420 study patients, 43 (10.2%) patients had MB. The clinical and demographic characteristics of the study population are summarized in Table 1. Compared to patients without AF, patients with AF had a significantly higher incidence of cerebrovascular disease (8.6% vs 1.9%; p = 0.001), were more symptomatic (the proportion of palpitation, 48.4% vs. 20%; *p* < 0.001), and had a higher prevalence of MB (19.0% vs. 7.3%; *p* = 0.001), PH (18.1% vs. 7.9%; p = 0.003) and hyperlipemia (39.0% vs 25.1%; p = 0.005). Moreover, left atrial diameter (49.9 ± 7.2 vs. 45.3 ± 7.0 *p* < 0.001) and interventricular septal thickness (20.4 ± 5.1 vs 19.2 ± 4.5; *p* = 0.024) were significantly greater in patients with AF than in those without AF. LVOT gradient was lower in patients with AF than in those without AF (77.9 ± 25.1 vs. 84.7 ± 28.1; *p* = 0.028).

**Table 1:** Baseline characteristics of the study population

Variables	HOCM with AF (N = 105)	HOCM without AF (N = 315)	*P*
Male (N, %)	62 (59.0)	188 (59.7)	0.909
Age (y)	50.7 ± 11.3	50.4 ± 11.2	0.779
BMI (kg/m^2^)	25.8 ± 3.9	25.6 ± 4.2	0.630
Heart rate (beats/min)	73.3 ± 14.0	71.8 ± 10.1	0.303
Systolic blood pressure (mmHg)	120.6 ± 14.6	122.8 ± 15.2	0.190
Diastolic blood pressure (mmHg)	72.7 ± 8.8	73.3 ± 9.3	0.573
Smoking (N, %)	39 (37.1)	122 (38.7)	0.840
NYHA class	2.65 ± 0.7	2.69 ± 0.5	0.329
Family history of HCM or SCD (N, %)	11 (10.5)	29 (9.2)	0.701
History of cerebrovascular disease (N, %)	9 (8.6)	6 (1.9)	0.001
*Concomitant disease*			
MB (N, %)	20 (19.0)	23 (7.3)	0.001
PH (N, %)	19 (18.1)	25 (7.9)	0.003
Hypertension (N, %)	27 (25.7)	94 (29.8)	0.419
Diabetes (N, %)	7 (6.7)	17 (5.4)	0.627
Hyperlipemia (N, %)	41 (39.0)	79 (25.1)	0.005
*Clinic presentation*			
Chest pain (N, %)	39 (37.1)	123 (39.0)	0.779
Amaurosis (N, %)	12 (11.4)	69 (21.9)	0.018
Palpitation (N, %)	51 (48.6)	63 (20.0)	0.000
Syncope (N, %)	18 (17.1)	73 (23.2)	0.194
*Echocardiographic indices*			
Left atrial diameter (mm)	49.9 ± 7.2	45.3 ± 7.0	0.000
LVEDD (mm)	42.3 ± 4.9	43.0 ± 4.8	0.198
IVST (mm)	20.4 ± 5.1	19.2 ± 4.5	0.024
LVOT gradient (mm Hg)	77.9 ± 25.1	84.7 ± 28.1	0.028
LVEF (%)	70.1 ± 6.3	70.0 ± 7.1	0.961
Moderate or severe MR (N, %)	44 (41.9)	140 (44.4)	0.650

Values are presented as mean ± SD or as N (%)

HOCM, hypertrophic obstructive cardiomyopathy; BMI, body mass index; NYHA, New York Heart Association; HCM, hypertrophic cardiomyopathy; SCD, sudden cardiac death; MB, myocardial bridging; PH, pulmonary artery hypertension; LVEDD, left ventricular end-diastolic diameter; IVST, interventricular septal thickness; LVOT, left ventricular outflow tract; LVEF, left ventricular ejection fraction; MR, mitral regurgitation

### Clinical data associated with AF

The variables associated with AF from the univariate logistic regression are presented in Table 2. After multivariate analysis, in addition to PH (OR 2.63; 95% CI 1.26–5.47; *p* = 0.01), hyperlipemia (OR 1.83; 95% CI 1.12–3.00; *p* = 0.016), left atrial diameter (OR 1.09; 95% CI 1.05–1.13; *p* < 0.001), interventricular septal thickness (OR 1.06; 95% CI 1.003–1.12; *p* = 0.037), and the presence of MB (odds ratio [OR] 2.33; 95% confidence interval [CI] 1.08–5.01; *p* = 0.03) were found to be an independent risk factors for AF.

**Table 2:** Conditional logistic regression models for analysis of the risk factors of AF

Variable	Univariate	*P*	Multivariate	*P*
	OR (95%CI)		OR (95%CI)	
Age	1.02 (0.97–1.06)	0.51		
MB	3.13 (1.57–6.12)	0.001	2.33 (1.08–5.01)	0.031
PH	2.55 (1.33–4.87)	0.005	2.63 (1.26–5.47)	0.01
Diabetes	1.24 (0.51–3.04)	0.633		
Hyperlipemia	1.85 (1.19–2.90)	0.008	1.83 (1.12–3.00)	0.016
Left atrial diameter	1.10 (1.06–1.13)	< 0.001	1.09 (1.05–1.13)	< 0.001
IVST	1.05 (1.01–1.10)	0.020	1.06 (1.003–1.12)	0.037
LVOT gradient	0.99 (0.982–0.999)	0.030		

OR, odds ratio; CI, confidence interval; MB, myocardial bridging; PH, pulmonary arterial hypertension; IVST, interventricular septal thickness; LVOT, left ventricular outflow tract

### Characteristics of MB in HOCM patients with AF and without AF

**Fig. 2:**
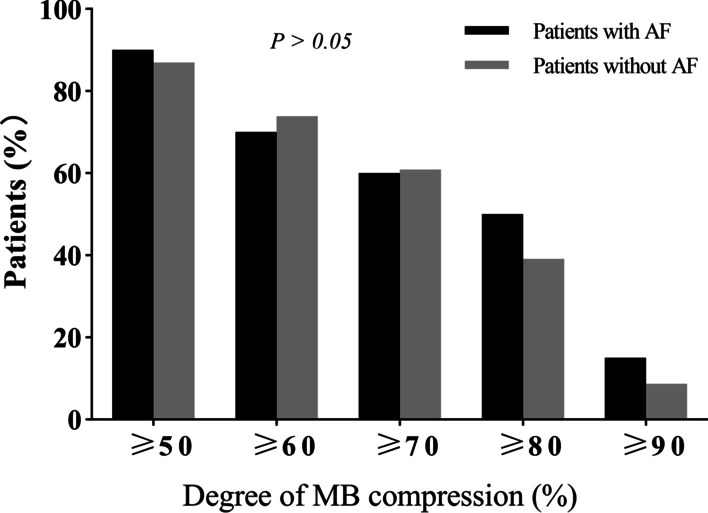
The proportion of different severity of MB compression in HOCM patients with and without AF

No difference was found between patients with and AF and those without AF in the proportion of different MB compression severity during systole (Fig. 1) and MB length on angiography (Fig. 2). Furthermore, we did not find any significant differences in the prevalence rate of MB, compression of MB during systole, and the length of MB on angiography between patients with paroxysmal AF and those with persistent AF (Table 3).

**Table 3:** MB characteristics in HOCM patients with paroxysmal AF and those with persistent AF

	HOCM with paroxysmal AF (N = 84)	HOCM with persistent AF (N = 21)	*P*
Prevalence rate of MB (N, %)	16 (19.0)	4 (19.0)	1.000
Compression of MB (%)	68.1 ± 19.7	65.0 ± 17.3	0.776
Length of MB (mm)	25.4 ± 6.0	19.5 ± 7.0	0.108

Values are expressed as mean ± SD or as N (%)

MB, myocardial bridging; HOCM, hypertrophic obstructive cardiomyopathy

## Discussion

**Fig. 3:**
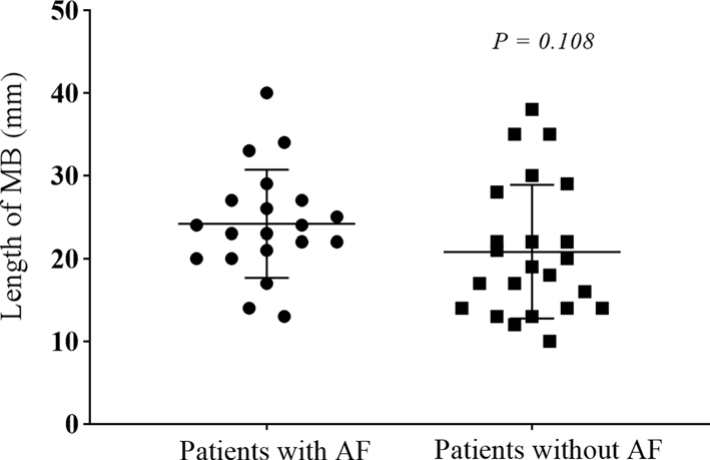
The length of MB on angiography between HOCM patients with and without AF

In this study, we reported a previously unknown association between MB and AF in patients with HOCM. The presence of MB was > twofold higher in patients with AF than in those without AF, although the degree of MB compression and the MB length did not differ between the two groups. Moreover, the presence of PH, higher prevalence of hyperlipemia, greater left atrial diameter, and greater interventricular septal thickness in the AF group, compared to the control patients, were also associated with AF. Despite these factors in the AF group, the presence of MB was found to be an independent predictor of AF after adjusting for these differences using multivariate analysis. These results suggest that the presence of MB was related to AF in HOCM patients, and the occurrence of AF was not merely associated with the length and compression of the MB (Fig. 3).

### Previous studies on the mechanisms of atrial fibrillation in HCM

Older age, greater left atrial diameter, and greater interventricular septal thickness were previously known to be associated with AF, and participated in the process of atrial stretch and remolding resulting in an increase in the dispersion of repolarization, thus potentiating the ability of ectopic triggers to maintain AF [5, 10]. Moreover, previous studies demonstrated that total cholesterol variability was associated with an increased risk of AF [11], and this supports our finding that a higher prevalence of hyperlipemia was an independent risk factor of AF. The potential link between hyperlipemia and AF has not been elucidated. However, there were two possible paths: in native atrial myocytes, cholesterol depletion increases membrane contents of rectifier K+ current, which is a primary repolarizing current in the human atrium and involved in the pathophysiological process of AF [12, 13]. Furthermore, cholesterol depletion impaired cardiomyocyte contractility, which may increase left ventricular end-diastolic pressure and left atrial pressure [14], leading to atrial remolding and precipitates the occurrence of AF.

Furthermore, PH also elevates right atrial pressure and results in electrical remolding, which increases the likelihood of AF [15]. In patients with PH, sinus recovery time is prolonged, atrial conduction slows with a significant reduction in conduction velocity, and increases activations times at a global and regional level [15]. Moreover, Kanbayashi et al. observed a higher prevalence of AF and systemic embolic events in patients with HCM with a PASP of > 40 mmHg during a 10.3 ± 7.4 years follow-up period. This finding was consistent with our finding that PH was linked with an increased risk of AF in patients with HOCM. This finding was consistent with ours that the presence of PH was linked with an increased risk of AF in patients with HOCM [16]. Similarly, in a 5-year prospective study involving 239 patients with PH or chronic thromboembolic pulmonary hypertension, Olsson et al. observed an incidence rate of new-onset AF or atrial flutter of 20%, and patients with PH seemed to have a higher risk of developing AF, compared with those with thromboembolic pulmonary hypertension [17]. Furthermore, patients with AF usually have an elevated left atrial pressure that leads to the development of post-capillary pulmonary hypertension and, in turn, aggravates PH, and this ultimately forms a vicious circle [18].

### The potential mechanism of MB and AF in HOCM patients

To the best of our knowledge, the presence of a MB alters coronary hemodynamics, leads to endothelial and microvascular dysfunction, and myocardial ischemia [19–23]. These changes caused by the MB could be responsible for the development of AF in patients with HOCM.

First, long-term myocardial ischemia is the main cause of myocardial fibrosis and a decline in ventricular function that increases left atrial and left ventricular end-diastolic pressures [5]. These changes can lead to atrial fibrosis, which may serve as an arrhythmogenic substrate for AF. A histological study demonstrated that patients with MB had a 33% increase in myocardial interstitial fibrosis compared with the control group [23]. In a three-dimensional speckle-tracking echocardiography study, the results indicated that left ventricular diastolic function, regional longitudinal strain, and area strain of the territory perfused by the MB reduced with increasing severity of systolic compression of the intramural coronary artery [24]. Furthermore, previous studies showed that ventricular fibrotic changes were more pronounced in AF patients than in subjects with sinus rhythm, and more extensive changes were found in patients with permanent or persistent arrhythmia compared to those with paroxysmal AF [25–27]. These results suggest that the presence of MB may play a role in the development of AF by increasing ventricular fibrosis and impairing ventricular function.

Additionally, coronary endothelial and microvascular dysfunction may act as potential pathophysiological substrates for AF. Corban et al. found that in patients with chest pain but no significant coronary stenosis, coronary endothelial dysfunction was associated with an increased risk of AF over a mean follow-up period of 10.5 ± 5.5 years [28]. Another study with a small sample size indicated that isolated atrial myocardial perfusion defects caused by microvascular dysfunction of the left atrial circumflex branch were associated with lone recurrent AF [29]. Our study also observed that patients with AF had a higher prevalence of MB, which was one of the causes of coronary endothelial and microvascular dysfunction. These findings strongly suggest that endothelial and microvascular dysfunction may participate in the process of AF. Further, previous studies also indicated that AF might impair endothelial dysfunction through multiple mechanisms such as altered hemodynamic and arterial shear stress on endothelial cells, decreased nitric oxide bioavailability, increased oxidative stress, and inflammation [30]. Thus, these results may suggest a bidirectional relationship between coronary endothelial dysfunction and AF, potentially forming a vicious cycle, which causes worse endothelial dysfunction and maintenance of AF.

To further illustrate the relationship between MB characteristics and AF, we compared the compression and length of MB between patients with AF and those without AF, as well as between patients with paroxysmal AF and those with persistent AF. Surprisingly, compression and the length of MB were comparable between patients with AF and those without AF and between patients with paroxysmal AF and persistent AF. To date, studies have demonstrated that many factors affected coronary hemodynamics of patients with MB, including the location, depth, and length of the bridging and the orientation of the bridging relative to myocardial fibers [31]. The effect of the length of MB on coronary hemodynamics has been controversial. Some studies have suggested that the length of an MB has a minor effect on coronary flow, while some studies have demonstrated that an increase in the length of the MB was associated with a decreased wall shear stress and increased blood residence time within the proximal segment [21, 32, 33]. In addition, the relationship between the degree of MB compression and coronary hemodynamics has not been clearly elucidated. Most studies show a direct relationship between the severity of MB compression and coronary hemodynamic perturbation in the proximal segment, and this is related to decreased wall shear stress and increased blood residence time [32, 34]. In our study, the depth and orientation of the bridging relative to myocardial fibers cannot be evaluated as the number of patients with MB was small. We could also not find a relationship between the characteristics of MB and AF. Further studies are needed to evaluate MB using a more appropriate methodology to identify the relationship between MB characteristics and AF.

In summary, the occurrence of AF may be aggravated by these pathophysiological changes through a synergistic effect. Nevertheless, the explicit mechanism of the link between MB and AF in patients with HOCM remains unclear; therefore, further studies and experiments are needed on this subject.

### Study limitations

The primary limitation of this study is the potential non-comparability of the patients with AF and the control group, and that could have been due to a selection bias. We attempted to address this issue in several ways. First, we used a 1:3 matched case–control study design to balance the distribution of age and gender between the two groups; however, having three controls per case made the results of the analysis more robust. Second, we adjusted for differences between the two groups using multivariate analysis. Although we used a matching technique and multivariate analysis to adjust for confounders between the two groups, our findings might be confounded by unmeasured covariates. Furthermore, due to constraints of the small sample size and MB evaluation method, we could not find a relationship between the anatomical characteristics of MB and AF. Moreover, this was a single-center study; therefore, these results should be cautiously generalize to other centers.

## Conclusion

This study demonstrated that the presence of MB-LAD was an independent risk factor for AF in patients with HOCM; however, we did not find a direct relationship between the length and compression of the MB and AF. Further studies are needed to focus on the evaluation of MB to identify the link between the features of MB-LAD and AF.

## Data Availability

The datasets generated and/or analysed during the current study are not publicly available due to Fuwai Hospital system, but data can be obtained from the corresponding author under reasonable request and with the permission of the Ethics Committee of Fuwai Hospital.

## Abbreviations

MB: Myocardial bridging
LAD: Left anterior descending
AF: Atrial fibrillation
HOCM: Hypertrophic obstructive cardiomyopathy
HCM: Hypertrophic cardiomyopathy
LVOT: Left ventricular outflow tract
PH: Pulmonary arterial hypertension
