# Correction to: Myocardial bridging of the left anterior descending coronary artery as a risk factor for atrial fibrillation in patients with hypertrophic obstructive cardiomyopathy: a matched case–control study

**DOI:** 10.1186/s12872-021-02204-1

**Published:** 2021-08-19

**Authors:** Changrong Nie, Changsheng Zhu, Qiulan Yang, Minghu Xiao, Yanhai Meng, Shuiyun Wang

**Affiliations:** 1 Department of Cardiovascular Surgery, Fuwai Hospital, National Center for Cardiovascular Diseases, Chinese Academy of Medical Sciences and Peking Union Medical College, Beilishi Road 167, Xicheng District, Beijing, 100037 China; 2 Department of Intensive Care Unit, Fuwai Hospital, National Center for Cardiovascular Diseases, Chinese Academy of Medical Sciences and Peking Union Medical College, Beijing, China; 3 Department of Ultrasound, Fuwai Hospital, National Center for Cardiovascular Diseases, Chinese Academy of Medical Sciences and Peking Union Medical College, Beijing, China

## Correction to: BMC Cardiovasc Disord (2021) 21:382 https://doi.org/10.1186/s12872-021-02185-1

Following publication of the original article [1], the authors identified an error in Fig. 1, in which the markers of “MB” should be marked on the left anterior descending coronary artery but were marked on the diagonal branch. This error does not affect the result and conclusion of this article. The updated Fig. 1 is provided in this correction article and the original article [1] has been corrected.

**Fig. 1:**
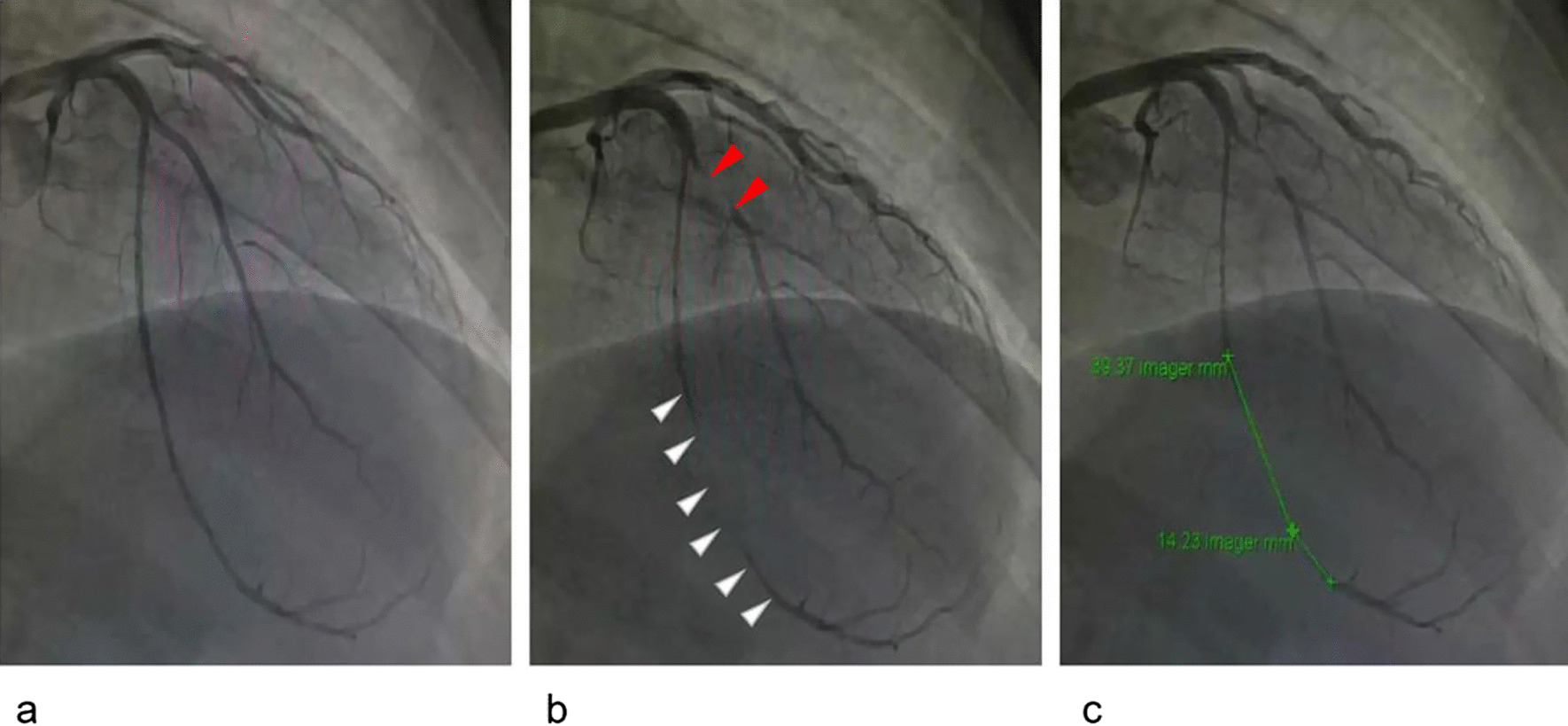
Myocardial bridging (MB) demonstrated by invasive coronary angiography during diastole (**a**) and systole (**b**). There was a significant narrowing on the anterior descending coronary artery during systole (white arrows). The MB on the diagonal branch (red arrows) or any other branch was rare and not analyzed in this study. The length from the beginning to the end of the coronary artery narrowing was measured as MB length (**c**)
